# Diet and Nutrition in Pediatric Inflammatory Bowel Diseases

**DOI:** 10.3390/nu13020655

**Published:** 2021-02-17

**Authors:** Ugo Cucinotta, Claudio Romano, Valeria Dipasquale

**Affiliations:** Pediatric Gastroenterology and Cystic Fibrosis Unit, Department of Human Pathology in Adulthood and Childhood “G. Barresi”, University of Messina, 98124 Messina, Italy; ugocucinotta@gmail.com (U.C.); dipasquale.valeria@libero.it (V.D.)

**Keywords:** diet, enteral nutrition, inflammatory bowel disease, nutrition, nutritional therapy, prevention

## Abstract

Both genetic and environmental factors are involved in the onset of inflammatory bowel disease (IBD). In particular, diet composition is suspected to significantly contribute to IBD risk. In recent years, major interest has raised about the role of nutrition in disease pathogenesis and course, and many studies have shown a clear link between diet composition and intestinal permeability impairment. Moreover, many IBD-related factors, such as poor dietary intake, nutrients loss and drugs interact with nutritional status, thus paving the way for the development of many therapeutic strategies in which nutrition represents the cornerstone, either as first-line therapy or as reversing nutritional deficiencies and malnutrition in IBD patients. Exclusive enteral nutrition (EEN) is the most rigorously supported dietary intervention for the treatment of Crohn’s Disease (CD), but is burdened by a low tolerability, especially in pediatric patients. Promising alternative regimens are represented by Crohn’s Disease Exclusion Diet (CDED), and other elimination diets, whose use is gradually spreading. The aim of the current paper is to provide a comprehensive and updated overview on the latest evidence about the role of nutrition and diet in pediatric IBD, focusing on the different nutritional interventions available for the management of the disease.

## 1. Introduction

The term inflammatory bowel disease (IBD) refers to a heterogeneous group of disorders encompassing Crohn’s disease (CD), ulcerative colitis (UC) and unclassified inflammatory bowel disease (IBD-U), characterized by a relapsing-remitting behavior, and variably presenting with abdominal pain, diarrhea, rectal bleeding and weight loss. In the last decades, the incidence of IBD has significantly grown in industrialized countries, in children as in adults [1,2,3]. It is well known that IBD has a multifactorial etiology, but despite the ongoing scientific efforts, pathogenesis and pathophysiology are still unclear [4]. Both genetic and environmental factors are involved in IBD onset. In particular, the prevailing hypothesis accounts a complex interaction between an exaggerated immune response in genetically predisposed individuals and environmental factors along with intestinal flora alterations, eventually sustaining an anomalous chronic inflammation [4,5].

Several studies have been conducted in the past to evaluate the precise role of nutrition (and malnutrition) and diet composition in immune-mediated diseases risk, leading to the development of a new discipline, referred to as “clinical nutrition”. Clinical nutrition is defined by European Society for Clinical Nutrition and Metabolism (ESPEN) as the “discipline that deals with the prevention, diagnosis and management of nutritional and metabolic changes related to acute and chronic diseases and conditions caused by a lack or excess of energy and nutrients” [6]. It encompasses all the diseases in which nutrition plays a main role, not only as a promoter, but also as a therapeutic tool [7]. For what IBD is concerned, the relation between diet composition and disease onset, course and management is supported by scientific evidence: i) epidemiological studies found an association between specific dietary habits and nutrients with an increased risk of IBD; ii) some foods and dietary components are potentially capable to either enhance or reduce the severity of inflammation; iii) for some pediatric patients with an established diagnosis of CD, exclusive enteral nutrition (EEN) can be considered a primary induction treatment with efficacy in achieving mucosal healing; iv) exclusion diets could treat or prevent disease flares; v) in IBD children, especially in CD children, malnutrition and nutrients deficiencies are often present at diagnosis; vi) early nutritional strategies can lead to a better disease control, as well as catch-up growth, bone mineral density improvement and adequate pubertal development [8,9,10]. The most common therapeutic choices for pediatric IBD consist of systemic and topical corticosteroids, amino salicylate and immunosuppressants (such as thiopurines or methotrexate). Starting from the past decade, use of biologic therapies has significantly increased for their clear efficacy also in pediatric setting, although they have been associated with a loss of response over time [11]. Beside this, non-pharmacological management of pediatric IBD has evolved over years and dietary changes are currently considered major therapeutic tools [10,12,13].

The aim of the present study is to provide a comprehensive and updated overview on the latest evidence about the role of nutrition and diet in pediatric IBD, also focusing on the different nutritional interventions available for the management of the disease.

## 2. Methods

For the purpose of the paper, a comprehensive search of the published literature through the databases PUBMED MEDLINE and GOOGLE SCHOLAR was performed. The following keywords: “diet”, “children”, “enteral nutrition”, “inflammatory bowel disease”, “clinical nutrition”, “nutritional therapy”, “malnutrition” were used. We focused on the most relevant articles published in English between 2005 and December 2020 including both Meta-analysis, Systematic Reviews, Consensus Guidelines, Randomized Controlled Trials and Cohort Studies. The selected papers were then analyzed in order to extrapolate valid and updated evidences about the role of nutrition and diet in pediatric IBD, with a focus on the nutritional strategies available for the management of the disease. 

## 3. Nutrition and Diet and Intestinal Inflammation

Despite the fact the exact pathogenesis is still unclear, IBD development results from a variable interaction between genetic susceptibility, individual immune response, gut microbiota and environmental factors [14]. 

The role of the diet composition in the intestinal inflammation has long been controversial, due to sometimes limited and conflicting data resulting from retrospective and case-control studies. However, it has been demonstrated that some individuals are more susceptible than others to develop the disease, depending on specific dietary practices. The increasingly “westernization” of lifestyle, mostly characterized by a high consumption of animal proteins and fats along with a poor intake of fruit, vegetables and fibers is associated with a higher risk of IBD [15,16]. Indeed, some epidemiological studies have demonstrated an increased risk of IBD among individuals moving from low-income countries, with low IBD incidence, toward high-income countries, hypothesizing that new environmental factors could increase the risk of the development of the disease [3]. While overall fat intake would not correlate with IBD, there is enough evidence to support that a low intake of omega-3 (*n*-3) and a high one of omega-6 (*n*-6) polyunsaturated fatty acids (PUFAs) is associated with an increased risk of CD. *N*-6 PUFAs (i.e., linoleic acid) are precursors to proinflammatory eicosanoids, whereas dietary *n*-3 PUFAs inhibits the formation of proinflammatory prostaglandins and leukotrienes through the arachidonic acid pathway, so their chronic imbalanced consumption may eventually lead to a pro- inflammatory state, oxidative stress and impaired intestinal mucosal permeability [17,18]. On the other side, a long-term intake of dietary fiber, particularly derived from fruit, has been associated with lower risk of CD, while no or conflicting association with UC has been found [19]. The most recent ESPEN guidelines recommend a diet rich in fruit and vegetables and low in *n*-6 PUFAs [20]. Western diet increases the risk of disease through food additives like emulsifiers and saturated fats, maltodextrins, carrageenin and carboxymethylcellulose, that have been associated with impaired intestinal permeability [21,22,23,24]. 

Even early life dietary patterns can be considered risk factors. Breast milk is rich in secretory IgA (sIgA), leukocytes and antimicrobial factors (lysozyme, lactoferrin, nucleotides), and promotes immune system maturation and T-lymphocytes pool creation, as well as immune response against infections [25,26,27]. It has been suggested that an early discontinuation of breastfeeding may facilitate the onset of several chronic conditions later in life, such as metabolic and autoimmune diseases. A recent meta-analysis comparing the exposure to breast milk among patients with CD and UC and controls confirmed an inverse association between breastfeeding and the risk of developing IBD [28]. Moreover, a dose depending effect has been hypothesized, with higher protection for longer-lasting breast milk exposure [28]. Additionally, since microbiome has been proven to differ according to the type of early diet, with relatively higher proportion of *Firmicutes* and *Actinobacteria* in breast-fed infants, it seems reasonable to assume that dysbiosis during the first months of life may negatively impact on immunological functions of the host microbiota [29,30,31].

Indeed, IBD has been consistently associated with gut dysbiosis, variably determined by specific foods and dietary habits. While commensal microbiome is physiologically abundant in *Firmicutes, Bacteroidetes, Actinobacteria* and *Proteobacteria*, IBD patients typically exhibit a low concentration of *Firmicutes*, such as *Bifidobacterium, Clostridia* and especially *Faecalibacterium prausnitzii*, whose protective role has been theorized for the possibility to stimulate anti-inflammatory cytokines (including IL-10) [12,32,33]. Conversely, increased concentrations of *Escherichia Coli* and other *Enterobacteriacee* has been found [32,33]. Even metabolic pathways are altered, because short chain fatty acids (SCFAs), such as acetate, propionate, and butyrate, normally produced by commensal bacteria through fermentation of food components, have been found reduced in IBD compared to controls. Further evidence includes reduced tryptophan metabolism and disrupted bile acids metabolism, with low bile acids production, classically associated with anti-inflammatory activities and T-cells regulation [33]. All these alterations eventually contribute to a decreased bacterial diversity, altered host barrier, increased permeability and subsequent intestinal inflammation.

## 4. Nutritional Strategies in Induction of Remission in Pediatric IBD

### 4.1. Exclusive Enteral Nutrition

Exclusive enteral nutrition (EEN) is the most important nutritional intervention in pediatric IBD, consisting of a complete liquid formula as the unique source of daily energy requirement for a period of 6-8 weeks [34]. Consensus guidelines of the European Society of Pediatric Gastroenterology, Hepatology and Nutrition (ESPGHAN) and European Crohn’s Colitis Organization (ECCO), as well as North American guidelines, recommend EEN as the first-line therapy for mild to moderate pediatric CD to induce remission both in the first flare-up and during relapses of symptoms [20,34,35,36,37]. The main indication for EEN is represented by active luminal CD, with solely inflammatory behavior (B1 according to Paris classification) and low-to-medium risk at diagnosis, regardless of the disease location (Table 1) [35].

By contrast, there are insufficient data supporting EEN use for extraintestinal manifestations or for perianal disease [36]. EEN advantages include: (i) high rates (up to 80%) of clinical remission; (ii) steroids sparing; (iii) correction of malnutrition and micronutrients deficiency; (iv) lean body mass increase; (v) improvement of growth and height Z scores; (vi) decreased longer term need for steroid and anti-TNF therapies; (vii) improvement of quality of life (QoL) [38,39,40,41]. Data supporting EEN in the management of UC is still lacking, and its use is still not recommended in these patients [36].

#### 4.1.1. EEN Efficacy

Many studies, including systematic reviews, meta-analyses and Cochrane reviews, proved EEN to be as effective as steroid therapy in inducing remission over a 6–8 week-period in CD pediatric patients, by inducing both mucosal and transmural healing [42,43,44,45,46,47]. The significant advantage of sparing steroids, traditionally used in the clinical setting, is represented by the avoidance of their short- and long-term side effects, such as poor growth, increased susceptibility to infections, and metabolic disorders (Cushing syndrome, osteopenia/osteoporosis, impaired glucose metabolism) [35,36,48,49]. Several trials have demonstrated EEN may be superior to steroids in CD children with active disease, for the achievement of mucosal healing with significant decrease of acute phase reactants, such as erythrocyte sedimentation rate (ESR), C reactive protein (CRP) and fecal calprotectin (FC) [50,51]. Particularly, FC is a reliable, non-invasive inflammatory marker and predictor of mucosal healing, and it has been observed to decrease up to 50% after the administration of EEN [52], although FC levels at the end of EEN regimen would not seem to predict the length of time until a future relapse [53]. Furthermore, a study examining FC changes after food reintroduction found that FC rises to pre-treatment levels within 4 months post-EEN, proving that a subclinical inflammation rapidly occurs after food reintroduction, with free-diet foods presumably acting as triggers of inflammation [54].

Although no predictive factors of EEN outcome are currently validated, disease severity and luminal disease seem to be the only predictors of EEN success, with mild to moderate disease [weighted Pediatric Crohn Disease Activity Index (wPCDAI) < 57.5, fecal calprotectin < 500 mcg/ g of stool, ileal involvement, CRP > 15 mg/L] and ileal disease having demonstrated a better response to EEN [55]. However, due to insufficient data supporting the association between efficacy of EEN and disease location [56,57,58], current guidelines do not recommend taking into account the disease phenotype when starting patients on EEN [20,35,36].

#### 4.1.2. Practical Issues of EEN

EEN is delivered through the administration of enteral nutrition (EN) formulas, available in a wide range of commercial formulations. These formulas are classified according to the protein source into intact proteins (polymeric), modified protein (elemental/semi-elemental) and disease specific formulas [39]. A nutritionally balanced polymeric formula should be the preferred choice in patients with intestinal sufficiency and in the absence of other medical conditions (e.g., co-existence of a cow’s milk allergy) [36]. Its optimal nutritional properties (approximately 45–60% carbohydrate, 15–20% protein, 30–40% fat), along with better palatability and lower price, makes polymeric feed the most commonly used [59]. However, despite these advantages, no EN formula has been demonstrated to be superior to others, with similar results in terms of achievement of clinical remission [42,58]. Oral route should be the preferred way, switching to nasogastric tube only in the case of inadequate oral intake [36,60]. Volumes are calculated according to the estimated average daily requirement, minding the caloric content of the chosen formula (standard concentration of 0.86-1 kcal/mL). Since resting energy expenditure has not shown to be increased in patients with CD during the different phases of disease, equations for estimating energy requirements can be used independently from the disease phase [60].

Despite the absence of evidence about the exact duration of an EEN regimen, the guidelines recommend a minimum of 6 weeks. Indeed, while symptoms may improve after few days and by week 3 in most cases [61], mucosal healing takes several weeks to occur. Food re-introduction is then performed over a period of 1 to 5 weeks, although no protocols for the proper management are currently available [36]. Notably, a a retrospective cohort study evaluating food re-introduction after a EEN cycle during 1 year of follow-up proved that rapid (3 days) food reintroduction after EEN is as effective as standard (5 weeks) food reintroduction, with no significant differences in clinical relapse rates: rapid 50% vs. standard 47% (p = 0.58) [62]. Further studies are warranted in order to assess the best timing in IBD pediatric population.

#### 4.1.3. Mechanism of Action of EEN

The exact mechanism through which EEN is capable of inducing mucosal healing is still unclear, but some evidence exists about the beneficial exclusion of specific dietary components, microbiome modulation, intestinal rest and direct anti-inflammatory effect of EEN [63,64]. Previously published trials have shown the ineffectiveness of partial enteral nutrition (PEN) associated with a free diet in inducing clinical remission, proving that dietary antigens, even in small quantities, are dangerous, thus suggesting that EEN could also work by excluding them [40,65]. After EEN administration microbiome composition has shown some modifications, including paradoxical reduction of microbial diversity and decreased proportion of presumably beneficial bacterial groups, such as *Bifidobacterium spp, Firmicutes* (including *Faecalibacterium prausnitzii*)*, Bacteroides/Prevotella* and *Proteobacteriaceae* [64,66,67]. In particular, a significant correlation was found between the decrease of *Bacteroides/Prevotella* group bacteria and the clinical improvement during EEN treatment [68]. Accordingly, regression of major EEN-induced microbiome changes after return to habitual free diet is well documented [67,68].

### 4.2. Partial Enteral Nutrition

PEN is a nutritional strategy based on the administration of a liquid enteral formula, not covering 100% of total energy requirements, along with whole foods. In different trials evaluating PEN for treatment of active CD or for maintenance of remission, the volume of formula has ranged from 35% to 90% of total energy requirements, while the foods consumed belonged mainly to free diet or to defined restrictive diets [24]. PEN in association with an unrestricted diet has not shown to be a good choice of treatment [65]. It has been found to be significantly less effective for the achievement of clinical remission than EEN or biologics [40], whereby its use is not currently recommended as induction strategy in active pediatric CD [35,36]. Further nutritional strategies combining PEN with specific exclusion diets are warranted. 

### 4.3. Crohn’s Disease Exclusion Diet

The Crohn’s Disease Exclusion Diet (CDED) is a validated dietary intervention, firstly conceived in 2014 by Sigall-Boneh and colleagues, combining PEN with a specific exclusion diet [69]. The rationale of CDED is the avoidance of certain foods and dietary components (such as additives like emulsifiers or maltodextrins, food preservatives, etc), mostly belonging to western diets, deemed to act as triggers for intestinal inflammation, dysbiosis, altered intestinal mucous layer and impaired barrier function [12,21,22,70,71]. In addition, the use of real foods, even though in a controlled way, seems to meet the request of patients and their parents, often blaming the monotony of EEN as responsible for a low adherence to treatment [72,73].

#### 4.3.1. CDED Efficacy

Sigall-Boneh and colleagues [69] first examined the effectiveness of CDED on 47 CD patients (mean age of 16 ± 5.6 years), through the administration of a polymeric formula covering 50% of their daily energy requirements and selected foods for the remaining 50%. After 6 weeks of treatment 70% of them (33/47) achieved clinical remission, evaluated through the Harvey-Bradshaw Index (HBI) and the PCDAI. Across the following 6 weeks, the quantity of polymeric formula was gradually reduced to 25%, while more selected foods were added to diet. Eighty percent of patients were still in remission at 12 weeks [69]. Another study demonstrated the efficacy of CDED in inducing remission even for children failing biological therapy [74].

A recent randomized controlled trial comparing CDED (50% PEN + CDED) to EEN in patients with luminal mild to moderate active CD demonstrated that CDED is as effective as EEN in inducing clinical remission at 6 weeks of treatment, with similar decrease in PCDAI and acute phase reactants [75]. Moreover, CDED seems to be even superior to EEN in terms of sustained remission rates and tolerability, leading authors to conclude that CDED + PEN is reasonably a good option either as first line treatment in luminal mild-moderate active CD or for maintenance of remission in the long term [75]. However, due to the lack of endoscopic assessment of mucosal healing - a major therapeutic goal - current guidelines still don’t recommend CDED + PEN neither as induction therapy nor for maintenance [35].

Another recent trial compared EEN to PEN plus a controlled diet rather similar to CDED to induce mucosal healing in active pediatric CD over a period of 6 weeks [76]. PEN group received a polymeric formula covering 75% of the daily requirement and one meal a day from an anti-inflammatory diet (AID) inspired to CDED (avoidance of processed foods with additives, animal fat, sugar, dairy products, and gluten) (Table 2). 

At week 6 clinical and endoscopic remission, defined as PCDAI <10 and Simple Endoscopic Score for CD (SES-CD) ≤ 2, respectively, were evaluated, showing similar rates of both between EEN and PEN, with higher rates of clinical remission in the PEN group (81.9%) than described so far in other trials evaluating PEN [34,40]. However, some limitations of the study should be taken into account. PEN group received a greater amount of EN formula (75% of the daily requirement) than in other studies on PEN, perhaps explaining the higher remission rates. Moreover, there was the lack of randomization, which may have affected the clinical evaluation. Additionally, a small number of patients (*n* = 11) completed the protocol. Before recommendations can be formulated, further studies are necessary, aimed to replicate these data and to evaluate the real efficacy on a larger sample size of patients. 

#### 4.3.2. Practical Characteristics of CDED

As previously mentioned, CDED owes its progressive diffusion both to the high efficacy in terms of clinical remission and to its variability, leading to a better acceptance by patients. CDED is a multi-stage whole food diet, based on the exclusion of animal and saturated fats, gluten, dairy products, or high-processed foods containing emulsifiers, and all packaged products. At the same time, it provides an increased consumption of fruits, vegetables, and resistant starch. The foods are classified into three main groups: mandatory foods, allowed foods and not allowed foods. CDED is then designed in 3 phases: the first two induction phases lasting 6 weeks each, while the third one starting from the 13^th^ week and referred to as “maintenance phase” [69,73]. During the first phase, 50% of nutritional requirement is given in the form of a polymeric formula, poor in lactose and fibers (e.g., Modulen, Nestlè), while the remaining 50% is supplied by mandatory foods (Table 3), source of high-quality proteins, pectin, resistant starch and other beneficial fibers, all necessary for the production of SCFAs [73,77].

During the second phase the percentage of calories provided by the liquid formula is lowered to 25%, more foods are permitted with higher inclusion of fruits and vegetables and little quantities of bread, red meat and legumes (found to potentially aggravate symptoms) [78]. The third and last phase still consists of 25% polymeric formula and does not have a specific duration, allowing the patient to continue with a controlled diet, rich in healthy foods, with the final aim of better controlling the disease in the long term [73].

### 4.4. Crohn’s Disease Treatment With Eating Diet

A newly devised nutritional intervention is the Crohn’s Disease Treatment With Eating Diet (CD-TREAT), an individualized diet that aims to replicate composition, and hence the effect on the microbiome, of EEN using ordinary foods [71,79]. As previously said, EEN is likely to work by exclusion of dietary components thought to be detrimental for gut function and microbiome composition. Hence, similarly to EEN, in CD-TREAT some specific components are excluded (like lactose, gluten, processed meat or some additives) while others are allowed (lean meats, fish eggs, some fruits and vegetables, rich in macronutrients, vitamins and fibers) (Table 4).

CD-TREAT strength is represented by the higher variability and palatability in comparison to EEN. It has shown good percentages of remission after 8 weeks both in adults and in children: 80% (4/5) of adults showed a clinical response (wPCDAI score change > 17.5), while 60% (3/5) of pediatric patients were in remission (wPCDAI score < 12.5) [79]. Despite the exiguous group of patients tested, which make it necessary to verify these on a larger sample of individuals, such a diet seems to be an encouraging step forward in the future approach to CD treatment, especially as a substitute to the long-term use of EEN, whose acceptability could be limited, and as a solid food-based alternative which would be preferable [72].

### 4.5. Further Nutritional Strategies

Further exclusion diets have been proposed, including the Specific Carbohydrate Diet (SCD), the Diet Low in Fermentable Oligo-, Di- and Monosaccharides and Polyol (FODMAPs), the Paleolithic Diet, and the Vegan Diet. Although some of them have shown interestingly results, their efficacy has been frequently evaluated in studies with several limitations. Many of these studies were mostly focused on the assessment of improvement in symptoms without standardized clinical outcomes, and with poor attention to inflammation and mucosal healing achievement [80]. Moreover, most data have been extrapolated from studies on adults, with sometimes conflicting results between those showing a real improvement of symptoms [81,82] and those showing no significative changing or even worsening of symptoms [83,84]. Moreover, some of these diets expose patients to the high risk of malnutrition due to the restrictiveness of foods allowed, so that their use is not routinely recommended in children/adolescents, unless potential benefits outweigh potential risks of the diet [20,36].

## 5. Nutritional Strategies in Maintenance of Remission in Pediatric IBD

Beyond the achievement of clinical remission, one of the most important goals in the management of IBD is the maintenance of remission, therefore avoiding, or at least delaying, the occurrence of a future relapse. Maintenance therapy of CD has classically consisted of immunosuppressants, such as thiopurines, or biologics in the last years. Despite their well-known efficacy, both of these treatments are burdened by some negative aspects, including risk of side effects for thiopurines (e.g., infections, pancreatitis), loss of response over time and high cost for biologics [35]. For these reasons, trials investigating nutritional strategies for maintenance of remission have been carried out in the last years. To date, EEN is not recommended for maintenance of remission in pediatric patients, since efficacy has not been thoroughly evaluated in children and long-term adherence may be challenging after a first cycle of induction [36,55,80]. Although other nutritional interventions are usually intended for short-period use, it is not infrequent for many patients to continue using them for longer period of time. This behavior derives, on the one hand, by the current absence of a recommended diet for IBD patients in the long term and, on the other hand, by the fear of relapses after food reintroduction. Indeed, many patients with an established diagnosis of IBD tend to avoid a variety of foods (most commonly grains, dairy products, vegetables and fruits) believed to provoke IBD symptoms and flares, or in order to ameliorate the control of disease [85,86]. Patients and their families frequently advocate dietary advices from physicians and are extremely interested about the proper dietary regimen to follow. 

According to the recent ESPEN guidelines [20], no specific diet needs to be followed during remission phases of IBD, since none of the proposed alternative diets seems effective in maintenance of remission. It is important to mention that most exclusion diets (e.g., FODMAPs, gluten-free diet, lactose free diet) may improve symptoms in IBD patients but haven’t been demonstrated to affect the inflammatory activity in the long term [9,36,71]. Diets should be then customized on the single patient according to individual’s preference or in case of specific intolerance (e.g., reducing high-lactose containing products and/or using lactase treated products if lactase deficiency is suspected), keeping in mind the risk of malnourishment or nutritional deficiencies [20].

A recent pediatric study demonstrated that a subgroup of patients, who achieved remission using EEN and not in therapy with any other medications, can successfully continue with PEN supplements for maintenance of remission with lower flares rates at 1 year of follow up [87]. By contrast, no significative differences in relapse rates have been observed between patients using PEN and azathioprine and those only using azathioprine. For this reason, PEN associated with exclusion diets have been proposed in case of patients with mild disease and who have a low risk of flares, despite amount, duration and timing of PEN is still unknown [36,88].

## 6. Malnutrition 

Malnutrition is a quite frequent occurrence in IBD patients, especially in CD patients, since different sites are affected in comparison to UC (small bowel vs colon-rectum). Pathophysiology of malnutrition is heterogeneous, involving multiple aspects, including: i) anorexia, food avoidance and self-imposed hypocaloric diets; ii) increased energy and nutrient losses, as a result of malabsorption and gut losses [89,90].

Data regarding prevalence of malnutrition in IBD children are mostly available among newly diagnosed patients, accounting approximatively 60% of newly diagnosed CD children and 35% of UC [90]. Protein-energy malnutrition is therefore a common finding in CD at the time of diagnosis, with low weight for age and body mass index (BMI). Then, nutritional status often fluctuates over time according to disease control and flares. Noteworthy, different studies have highlighted a progressive decrease in the degree of malnutrition among newly diagnosed IBD patients in recent years, especially in UC patients, in whom even overweight has been observed [90]. 

Nutritional assessment is a central component in the management of IBD patients, since a poor nutritional status is strictly related to growth failure, poor bone accrual, anemia, disrupted pubertal development over time and short stature in adulthood, as well as increased complications rates and poor prognosis [36,91,92]. Recommended approach to IBD patients should comprise: i) a periodical dietary history through a 3- to 5-day record of qualitative and quantitative intake of foods (macro and micronutrients); ii) evaluation of nutritional status with assessment of weight-for-age, height-for-age, and BMI z-score at every visit, and assessment of height velocity every 6 months [36,91].

## 7. Micronutrient Deficiency

Micronutrients and vitamins are frequently lacking in IBD, due to malabsorption linked to inflammation and/or to sub-optimal nutritional intake. Micronutrients deficiency can be present at the diagnosis as well as develop during the clinical course of the disease, so that IBD patients should be checked for micronutrients levels on a regular basis, and specific deficits should be appropriately corrected [92,93,94]. The most frequent vitamin deficiencies observed in IBD-patients are water-soluble B9 and B12 deficiencies, physiologically absorbed in the duodenum, jejunum and ileum, which are frequent site of inflammation [90,95]. About micronutrients, iron and zinc are the most frequently lacking, with iron-deficient anemia (IDA) being the predominant type [90,96,97].

Necessity for single nutrients intervention varies according to type of elements/minerals considered. Zinc, selenium and magnesium deficiency should be not routinely assessed or supplemented [36]. By contrast, IDA should be periodically ruled out, especially during period of active inflammation. Treatment varies according to the disease phase: oral iron supplementation can be the choice of treatment in case of controlled disease and hemoglobin level > 10 g/dl, always keeping in mind the high risk of intolerance for the oral route. In case of active disease or moderate-severe anemia (hemoglobin < 10 g/dl), intra-venous ferric carboxymaltose replacement should be preferred [36]. Both children and adolescents are at risk of vitamin D deficiency during the clinical course of disease and should be always investigated for it. Vitamin D is a risk factor for low bone mineral density, whose additional risk factors are represented by cumulative corticosteroid dose, height-for-age Z-score, and BMI Z-score [20,96]. For this reason, according to current guidelines, all children affected by CD and steroid-treated, serum calcium and 25(OH) vitamin D should be monitored and supplemented, if required, to help preventing low bone mineral density. Vitamin D level below 50 nmol/L or 20 ng/mL suggests vitamin D deficiency, requiring a proper supplementation. If encountered, osteopenia and osteoporosis should be then managed according to current osteoporosis guidelines [20]. For the risk of folate (B9) deficiency, especially in those using sulfasalazine and methotrexate, its measurement is generally recommended annually, with folate supplementation (1 mg/day or 5 mg/week) in case of documented deficiency [36].

## 8. Conclusions

Dietary factors and malnutrition play a primary role in the onset and management of pediatric IBD. An appropriate diet can decrease the risk of IBD flares, and an age-adequate nutritional status can decrease the risk of complications and surgery in the long-term stage of the disease. Enteral nutrition has a significant impact on mucosal inflammation in CD, and clinical response to oral polymeric diet is associated with down-regulation of mucosal pro-inflammatory cytokines. Exclusive enteral nutrition or CDED diet have shown to be more effective than steroids in inducing clinical remission in pediatric patients. 

## Figures and Tables

**Table 1:** Summary of EEN characteristics.

Indications (according to Paris Classification at Diagnosis)	B1 (Inflammatory)
Advantages	High rates of clinical remission and mucosal healing Increased steroid-free remission Avoidance of steroids related side-effects Treatment of malnutrition and nutrients deficiencies Adequate growth and better QoL
Disadvantages	Low palatability No other food allowed High risk of early withdrawal High cost related to elemental diet Possible side effects (mostly diarrhea and vomiting)
Route of administration	Oral (preferred choice) Nasogastric tube
Duration of treatment	Minimum of 6 weeks up to 12 weeks
Type of formula	Polymeric or elemental

EEN, Exclusive Enteral Nutrition; B1, non-stricturing non-penetrating Behavior; QoL, Quality of Life.

**Table 2:** Not allowed foods in all phases - adapted from Sigall-Boneh et al. [69].

- Dairy products of any kind, margarine - Wheat, breakfast cereals, breads and baked goods of any kind, yeast for baking - Gluten-free products not listed above, Soya products, potato or corn flour - Processed or smoked meats and fish (sausages, luncheon meats, salamis, fish sticks) - Sauces, salad dressings, syrups and jams of any kind - Canned products and dried fruits - Packaged snacks (potato chips, pretzels, popcorn, nuts, etc) - All soft drinks, fruit juices and sweetened beverages, alcoholic beverages, coffee - Candies, chocolates, cakes, cookies and gum

**Table 3:** Example of Crohn Disease Exclusion Diet - adapted from [70,73].

Phase	Polymeric formula	Mandatory foods	Allowed foods
1 (Week 1–6)	Modulen ^®^ (50% of daily requirements)	Fresh Chicken breast (150–200 g/d) 2 Eggs/d 2 Bananas/d 1 Fresh Apple/d 2 Potatoes/d	Fresh Strawberries Fresh Melon (1 slice) Rice flour White rice and rice noodles (unlimited) 2 Tomatoes (additional allowed for cooking) 2 Cucumbers (medium size) 2 Avocado halves 1 Carrot Spinach 1 cup uncooked leaves Lettuce (3 leaves) Onion Fresh green herbs (basil, parsley, coriander, rosemary, thyme, mint, dill) 1 glass of squeezed orange juice from fresh oranges Water, sparkling water Salt, pepper, paprika, cinnamon, cumin 3 tablespoons honey 4 teaspoons sugar Fresh ginger and garlic cloves, lemons
2 (Week 7–12)	Modulen ^®^ (25% of daily requirements)		
3 (Maintenance) (Week 13 onwards)	Modulen ^®^ (25% of daily requirements)	No mandatory food	

**Table 4:** Exclusion diets with related included and excluded food groups - adapted from [71].

Dietary intervention	Excluded	Included
Crohn’s Disease exclusion diet	Dairy, gluten, processed meats, animal fat, canned and packaged foods, coffee, alcohol, emulsifying agents	Selected fruits and vegetables, fish, eggs, lean meats
Crohn’s Disease Treatment With Eating Diet	Lactose, gluten, processed meats, animal fat, alcohol, emulsifying agents	Fruits and vegetables, high fiber content, lean meats. Increased macronutrients, vitamins, minerals (aim to mimic EEN diet)
IBD Anti-InflammatoryDiet	Most dairy, gluten-based grains, fruits with seeds, refined sugars, processed foods, trans-fatty acids	Most fruits and vegetables, soluble fibers, flax meal, chia seed, oats, some type of yogurts and cheeses, nut and legume flours, eggs, fish, lean meats
